# Life-threatening duodenal perforation complicating endoscopic retrograde cholangiopanceatography: A case series^star;^

**DOI:** 10.1016/j.ijscr.2020.01.001

**Published:** 2020-01-14

**Authors:** Hussam M. Mousa, Ashraf F. Hefny, Fikri M. Abu-Zidan

**Affiliations:** Department of Surgery, College of Medicine and Health Sciences, UAE University, Al-Ain, United Arab Emirates

**Keywords:** Duodenum, Endoscopic, Retrograde, Cholangiopanceatography, Perforation

## Abstract

**Introduction:**

Endoscopic retrograde cholangiopancreatography (ERCP) is a diagnostic and therapeutic procedure for biliary and pancreatic diseases. It is associated with low rate of complications. However, some complications as duodenal perforation can be fatal.

**Presentation of cases:**

852 patients underwent ERCP at our hospital, six patients had a duodenal perforation (0.7 %). All patients were admitted with clinical and biochemical findings of obstructive jaundice without acute cholangitis. All patients had biliary tree dilatation confirmed on abdominal ultrasound scan and/or magnetic resonance cholangiopanceatography. Two patients were initially managed surgically, one of them died due to multi-organ failure. The other four patients were initially treated conservatively; two of them failed conservative management with one death due to sepsis, other two patients recovered without complications. The overall mortality rate was (33.3 %).

**Discussion:**

Multiple attempts of CBD cannulation and pre-cut sphincterotomy may increase the possibility of duodenal perforation. In the presence of clinical suspicion of perforation, an early radiological imaging is helpful for an early intervention.

**Conclusion:**

A high index of suspicion is essential for early diagnosis and intervention to improve the clinical outcome. In difficult ERCP, performing a post-procedural fluoroscopy study with contrast injection is essential.

## Highlights

• ERCP related duodenal perforation is rare but it is associated with high mortality.
• Early diagnosis of Post ERCP duodenal perforation is essential in reducing morbidity.
• A final fluoroscopy images of the biliary tree is helpful in the early detection of the perforation.

## 1 Introduction

ERCP is a diagnostic and therapeutic procedure. It has a low rate of complications. However, some of them can be fatal. Sphincterotomy is technically challenging [1]. It is usually performed after biliary cannulation under fluoroscopy guidance. Pre-cut sphincterotomy is a risky technique as the incision is made on the papilla before biliary cannulation. It requires special skills with a debate regarding its efficacy, timing, and complications [1].

Duodenal perforation complicating ERCP is uncommon. It is estimated to be between 0.1 and 1 % [2,3], having a mortality of 16–18 % [[3], [4], [5]]. Traditionally, many prefer an early surgery so as to avoid intra-abdominal sepsis [3]. However, conservative management has a role mainly in managing clinically stable patients despite radiological evidence of perforation [3,6].

We aim to study the incidence, time to diagnosis, management, morbidity, and mortality of post ERCP duodenal perforation at our institution to improve the clinical outcome of the patients.

## 2 Presentation of cases

All Patients who had post-ERCP duodenal perforation at our institution between April 2007 and December 2017 were retrospectively studied. Analyzed variables included; indications for ERCP, ERCP procedural difficulties, clinical presentation of post-ERCP duodenal perforation, diagnostic methods, time to diagnosis, management, length of hospital stay, and clinical outcome.

**Fig. 1:**
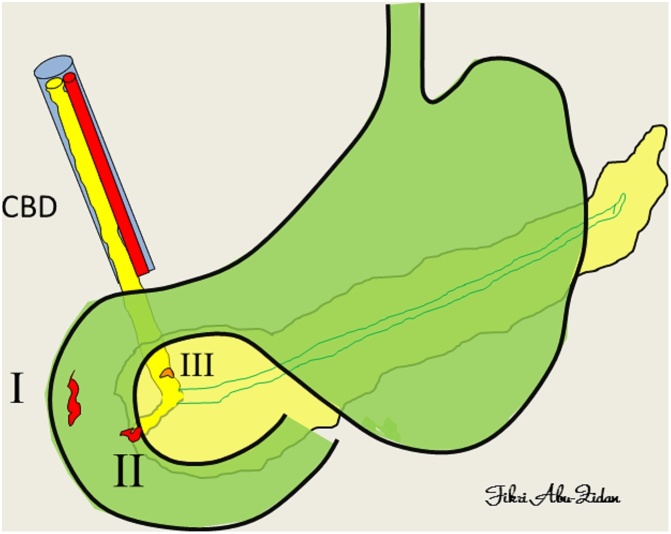
A diagram showing the four types of ERCP-related duodenal perforations: lateral or medical duodenal wall (type I), peri-ampullary (type II), and distal common bile duct (CBD) injury (type III). Type IV is the presence of retroperitoneal air without gross injury. (classification of Stapfer et al., 2000).

According to Stapfer et al. duodenal perforation was classified as follows; Type I is a lateral or medial duodenal wall perforations, Type II is a peri-ampullary injury, Type III represents distal bile duct injury and usually is caused by wire or basket instrumentations, and Type IV which is the presence of retroperitoneal air (RPA) alone [3]. Type IV is related to the amount of air insufflated and is usually treated non-surgically [3,[7], [8], [9]] (Fig. 1).

### 2.1 Indications for ERCP

During the period between April 2007 and December2017, 852 ERCP procedures were performed in our institution. Six patients (five females and one male) (0.7 %) had an ERCP- related duodenal perforation. The patients had a median (range) age of 45 (25–87) years. All patients were admitted with clinical and biochemical findings of obstructive jaundice. There was radiological evidence of biliary tree dilatation in all patients (Table 1).

**Table 1:** ERCP-related duodenal perforations treated at Department of Surgery, Al-Ain Hospital, Al-Ain, UAE, 2007–2017, (n = 6).

Patient #	Age/ Gender	ERCP difficulties	ERCP findings	ERCP duration (minutes)	CT Scan findings	Time to diagnosis (hours)	WBC	CRP	Perforation type	Management	HS (days)	Outcome
1	52/M	Stents placement	CHC	126	RPA	20	13.1	65.5	IV	Non-surgical	18	Recovery
2	87/F	Duodenal diverticulum	CHC	97	IPA, RPA	Immediate	11.1	83.2	I	Non-surgical	50	Died
3	42/F	Bleeding	CHL	58	Not done	24	20	6.3	II	Surgical	23	Died
4	35/F	PCS	Failed	91	RPA, No leakage Pneumothorax,	3.5	10.1	–	III	Non-surgical	12	Recovery
5	48/F	PCS	Failed	90	Emphysema, contrast leak on FU CT scan	11	18.8	355	I	Non-Surgical/ Surgical	22	Recovery
6	25/F	PCS	Sludge	95	IPA, RPA, Pneumothorax	13.5	9.1	121	I	Surgical	13	Recovery

**ERCP**: Endoscopic retrograde cholangiopancreatography; **M**: male; **F**: female; **OJ:** Obstructive jaundice; **CHL**: choledocholithiasis; **CHC:** cholangiocarcinoma; **PCS**: Precut-Sphinctorotomy; **RPA**: Retro peritoneal air; **IPA**: Intra-peritoneal air; **WBC**: white blood cells; **CRP**: C reactive protein; **HS**: Hospital stay; **FU:** Follow up.

### 2.2 ERCP procedure

All patients underwent ERCP with sedation in a prone position. The median (range) time for ERCP was 93 (58–126) minutes. Complete ERCP procedure including cannulation, sphinctorotomy; with or without common bile duct (CBD) stent insertion was achieved in only two patients. Four patients had difficult cannulation. Pre-cut sphinctorotomy was performed in three patients while the fourth had perforation at the site of a duodenal diverticulum identified during the procedure. As a result, ERCP was abandoned.

Two of the three pre-cut sphinctorotomy patients had failed re-cannulation (Table 1).

### 2.3 Time to diagnosis

**Fig. 2:**
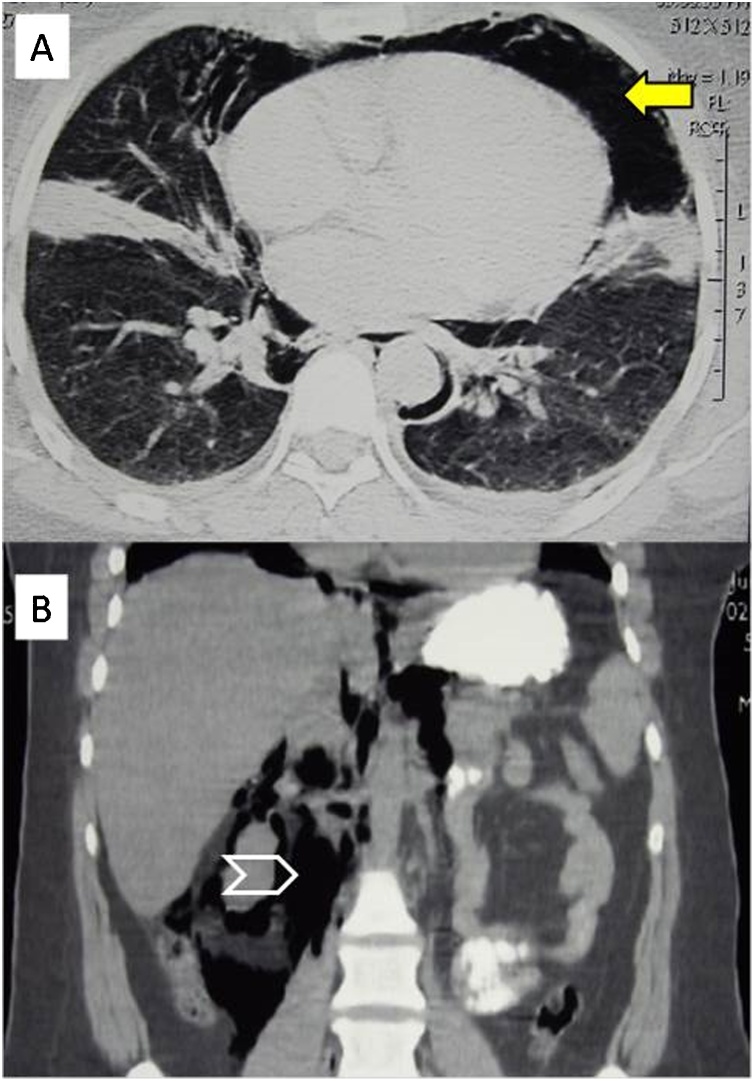
A 35-year-old woman presented with hypochondrial pain. Abdominal ultrasound showed thickening of the gall bladder wall and a dilated common bile duct of 8−10 mm. ERCP was tried but failed. The patient developed consistent upper abdominal pain following the ERCP. Chest CT scan (A) and coronal reconstruction of the abdominal CT scan (B) showed left pneumothorax (arrow) and retroperitoneal air (arrowhead).

All patients had abdominal pain and tenderness. Table 2 shows a comparison between the early (within 12 h) and late (12−24 h) clinical findings of the same patients. Perforation was discovered during the ERCP in one patient (16.6 %). In the remaining five, the median (range) time to diagnosis was 13.5 (3.5–24) hours. Following the persistence of abdominal findings, five patients (83.33 %) underwent abdominal CT Scan with oral contrast as the main diagnostic method (Fig. 2). Retroperitoneal air was detected in all five patients, two of whom were found to have surgical emphysema, and both underwent surgery. Two patients had pneumothorax on the right side.

**Table 2:** Early and late clinical findings of patients with post ERCP duodenal perforation (n = 6).

Clinical findings	Early (within 12 h) Number %	Late (12–24 h) Number %
Abdominal Pain	6 100	6 100
Abdominal distension	4 66.6	5 83.3
Tenderness	6 100	6 100
Guarding	5 83.3	5 83.3
Rebound Tenderness	2 33.3	4 66.7

### 2.4 Assessment of outcomes

Two patients (33.33 %) were hemodynamically stable with no leakage of oral contrast on abdominal CT Scan. Both were treated conservatively and were discharged home in a good condition.

One elderly woman with significant cardio-pulmonary and renal comorbidities had perforation at the site of a duodenal diverticulum. She was treated non-surgically due to her comorbidities following the failure of closure attempts using endoscopic clips. She died on the 50th day with multi- organ failure.

One patient had failed the non-surgical treatment. She developed peritonitis with leakage of oral contrast on the follow up CT Scan. She underwent laparotomy, primary closure of the perforation. Her post-operative period was uneventful.

Two patients (33.3 %) were haemodynamically unstable. Their abdominal CT Scan showed free retro-peritoneal air. Both were treated surgically. The first patient underwent multiple laparotomies as she had significant intra peritoneal bleeding with perforation in the distal CBD. This patient died on day 23 after admission due to multi-organ failure and sepsis. The second patient was found to have type I perforation treated with primary closure with optimum outcome. Both patients who had a pneumothorax were treated with chest tube insertion. Overall two patients died (mortality rate of 33.3 %).

## 3 Discussion

Our study has shown that the incidence of ERCP-related duodenal perforation was 0.7 % which is similar to others [2,7]. However, mortality rate was 33.3 % which is higher than the international incidence rate (8–23 %) [4,5,8]. This could be explained by late diagnosis and consequently the delay in the management. In the present study, the average time to diagnose duodenal perforation was 12 h. In another study, the groups with good clinical outcomes were significantly diagnosed early (0 h) compared with groups with poor outcomes (14.5 h) [2].

The diagnosis of type I duodenal perforation can be made during ERCP in 73 % of cases [10]. In the current study, only one patient out of three who had type I perforation was recognized during the procedure (33.3 %). Delay in identification and intervention of type I duodenal perforation is associated with high morbidity and mortality [4,5,8,9]. Types II–IV duodenal perforations are difficult to identify compared with type I which is caused by the endoscope [7].

Pre-cut sphincterotomy is a risk factor for post-ERCP perforation [11]. Generally, it is applied after many attempts of cannulation, which may lead to trauma and ampullary edema [11,12]. Sundaralingam et al. indicated in a meta-analysis that early pre-cut sphincterotomy significantly reduces post ERCP perforation compared with the standard technique [12].

The clinical diagnosis of post-ERCP perforation is difficult in the early stages because abdominal pain is a common finding. Peritonitis is usually a late manifestation; hence, the absence of peritonitis does not exclude a retroperitoneal perforation. Peritonitis is an indication for surgery [2]. Inflammatory markers, although alarming, did not have a role in determining the type of treatment [3]. In the presence of clinical suspicion of perforation, it is recommended to perform thoraco-abdominal CT scan with oral contrast.

Retroperitoneal air (RPA) is present in all types of post-ERCP perforations. Nevertheless, it is not an absolute indication for surgery [4]. In our study RPA was found in all five patients who had CT scan, two of them were managed non-surgically. The amount of RPA does not indicate the size or the seriousness of perforation; it is likely related to the amount of air insufflated in a protracted procedure [4,11]. Surgical emphysema can be an alarming sign for the need for early surgery. In our study, the two patients who had surgical emphysema needed surgery. The leaking air through the crura of the diaphragm may cause tension pneumothorax that must be suspected when there is unexplained chest pain, hypotension and dyspnea following an ERCP [13].

Confirmed type I duodenal perforation, peritonitis and significant contrast leakage on upper gastrointestinal fluoroscopy or abdominal CT scan are clear indications for early surgical intervention [5,14]. In our study, surgical intervention was indicated in patients with hemodynamic instability, peritonitis, and/or contrast leakage on the initial or the follow up CT scan. Non-surgical management could be applied in type II and III perforations in stable patients without peritonitis [6,14].

The presented article has been reported in line with the updating consensus preferred reporting of case Series in Surgery (PROCESS) Guidelines [15].

## 4 Conclusion

We recommend performing a final image with contrast injection through the endoscope to the duodenum for the early diagnosis of duodenal perforation especially in difficult ERCP procedures. Despite the rarity of this complication, it has a high morbidity and mortality. Multidisciplinary approach is needed to optimize the clinical outcome of this serious complication.

## Funding

Self-funded.

## Ethical approval

Approval has been given by Al Ain Hospital Research Governance Committee, Al Ain, UAE, has approved this study. (Ethical approval Number: AAHEC-2-18-074).

Our study has been registered with Research Registry. The unique identifying number is: researchregistry5269.

## Consent

Written informed consent was obtained from the patient's guardian / from the patient for publication of this case series and accompanying images. A copy of the written consent is available for review by the Editor-in-Chief of this journal on request.

All patients admitted to Al Ain Hospital give informed consent and agreement to participate in research and publications, and ethical approval was issued accordingly.

## Author contribution

Hussam MM: study concept, data collection, interpretation, writing the first draft, editing the paper, and approved the final version.

Hefny AF: study concept, interpretation, help writing first draft, editing the paper, and approved the final version.

Fikri MA: study concept, interpretation, editing the paper, and approved the final version.

## Registration of research studies

Not applicable.

## Guarantor

All the authors are responsible for the article.

## Provenance and peer review

Not commissioned, externally peer-reviewed.

## Declaration of Competing Interest

There is no conflict of interest among all the authors
